# A core outcome set for all types of cardiac surgery effectiveness trials: a study protocol for an international eDelphi survey to achieve consensus on *what* to measure and the subsequent selection of measurement instruments

**DOI:** 10.1186/s13063-015-1072-8

**Published:** 2015-12-02

**Authors:** A. Moza, C. Benstoem, R. Autschbach, C. Stoppe, A. Goetzenich

**Affiliations:** Department of Thoracic and Cardiovascular Surgery, University Hospital RWTH Aachen, Pauwelsstrasse 30, 52074 Aachen, Germany; Department of Anaesthesiology, University Hospital RWTH Aachen, Pauwelsstrasse 30, 52074 Aachen, Germany

**Keywords:** Core outcome set, COS, Domains, Cardiothoracic surgery, Clinical trials, Effectiveness, Health interventions

## Abstract

**Background:**

Cardiovascular disease (CVD) is a major contributor to the burden of disease and the number one cause of death worldwide. From 1990 until today, more people died from coronary heart disease than from any other cause. CVD is regularly treated with minimally or non-minimally invasive off- or on-pump cardiothoracic surgery and several interventions related to the outcome of the surgical procedures have been evaluated in clinical trials, but heterogeneity in outcome reporting hinders comparison of interventions across trials and limits the ability of research synthesis. This problem is encountered with the introduction of core outcome sets (COSs), which should be measured and reported, as a *minimum*, in all clinical trials for a specific clinical field.

**Methods/design:**

This study protocol describes the methods used to develop a COS for all types of cardiac surgery effectiveness trials. We aim to reach consensus on *what* to measure in an international three-round eDelphi exercise involving adult patients in need or after cardiothoracic surgery, cardiothoracic surgeons, cardiologists, anaesthesiologists, nursing staff and researchers with expertise in this particular field of medical research. Subsequently, outcome measurement instruments (how to measure) will be determined. Recommendations on COS development given by the Core Outcome Measures in Effectiveness Trials (COMET) Initiative and the Outcome Measures in Rheumatology (OMERACT) Initiative were followed.

**Discussion:**

The proposed COS aims to provide methodological guidance for future cardiothoracic surgical trials to ensure the comparability of effects of interventions across studies and enable research synthesis. This does not imply that primary outcomes should always and exclusively be those of the COS. However, to ensure the comparability of results across trials, the outcomes included in this COS should be considered for inclusion besides measuring trial-specific clinical endpoints.

## Background

Cardiovascular disease (CVD) is the number one cause of death worldwide and therefore, the major contributor to the burden of disease. Since 1990, more people have died from coronary heart disease than from any other cause. In 2008, 30 % of all global deaths (17.3 million) were attributed to CVD [1]. Of these deaths, an estimated 7.3 million were due to coronary artery disease [2]. CVD results from blockage of the coronary arteries by atherothrombosis, which is regularly treated with minimally or non-minimally invasive off- or on-pump cardiothoracic surgery.

A wide range of health interventions related to the outcome of cardiac surgery has been evaluated in clinical trials. At present, more than 3000 clinical trials investigating CVD are listed in the International Clinical Trials Registry Platform of the World Health Organisation [3] involving thousands of patients and costing millions in research funding. The results of clinical trials assessing similar interventions are summarised in systematic reviews and meta-analyses intending to provide the basis for clinical practice guidelines and treatment recommendations. However, it has been reported that clinical endpoints in cardiothoracic interventional research are measured and reported inconsistently [4], limiting the ability of research synthesis, a problem that is well known to systematic reviewers [5]. As a consequence, a significant number of identified matching studies are regularly excluded from meta-analyses, reducing their power and limiting the value of available evidence [6]. Furthermore, empirical research strongly determines that outcome-reporting bias (defined by the Cochrane Collaboration as “selective reporting of some outcomes but not others, depending on the nature and direction of the results”, Cochrane Handbook, Chapter 10, Table 10.1.a) has a significant impact on how the results of clinical trials are reported [7], strongly influencing the recommendations given by systematic reviewers.

This problem is encountered with the introduction of minimum core outcome sets (COSs) for a specific clinical field [5, 8]. A minimum COS is defined as an agreed minimum set of outcomes that should be measured and reported in all clinical trials for a specific clinical area [5]. COSs are intended to increase the reporting of outcomes important to all stakeholders, limit study heterogeneity and avoid selective reporting [8].

**Fig. 1:**
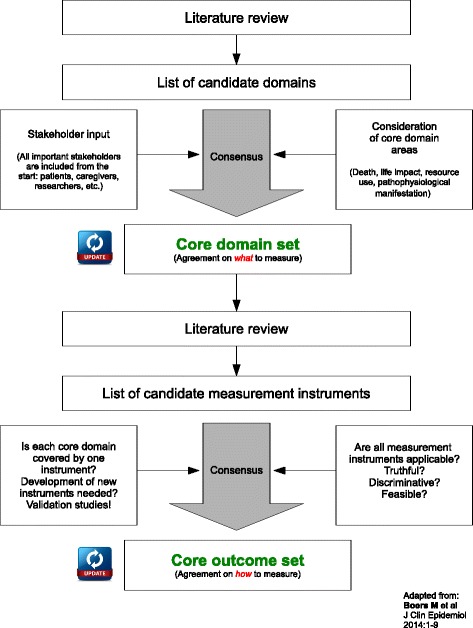
Development of a COS

The Outcome Measures in Rheumatology (OMERACT) Initiative [9] suggests a stepwise approach (Fig. 1) to COS development and introduced a new framework, which aims to include all key aspects of a health condition to ensure the comprehensiveness and applicability of COSs [9]. OMERACT Filter 2.0 (Table 1) was created to broaden the international classification of functioning, disability and health [10] and suggests four core domain areas: (1) death, (2) life impact, (3) resource use and (4) pathophysiological manifestations. By doing so, the outcome domains and measurement instruments will be consistent with the reporting framework currently introduced for the US Clinical Trials Registry [11] and will completely cover what is measurable in a clinical trial comprising both patient-centred and intervention-specific results.

**Table 1:** OMERACT Filter 2.0 framework: core areas to be considered for outcome measurement in health intervention studies (adapted from Boers et al. [9])

Core area	Specification
Death	This core area includes possible specifications of death, such as generic or disease-specific (all causes versus disease-specific mortality), and intervention-specific (for example, death due to surgery).
Life impact	This core area can include domains of the International Classification of Functioning, Disability and Health (ICF) (such as activity and participation) and domains for health-related quality of life (such as functional status, general health perceptions and overall quality of life).
Resource use and economic impact	This core area describes the economic impact of health conditions both on society and on the individual. A health condition and its treatment incur resource use.
Pathophysiological manifestations	This core area assesses whether the effect of the intervention specifically targets the pathophysiology of the health condition. Pathophysiology can include psychosocial manifestations. Example domains are ICF body function, reversible manifestations (including modifiable risk factors and actual manifestations of ill health) and irreversible manifestations (including unmodifiable risk factors and damage). This area can also encompass all biomarkers and surrogate outcomes.

Experts in the field have agreed that a COS would enhance the reliability of systematic reviews [12]. However, a recently published systematic review [13] on available COSs for comparative effectiveness research highlighted that no COS exists for trials investigating pre-, intra- or post-surgical interventions in conventional cardiac surgery (elective and emergency non-minimally invasive off- or on-pump procedures, excluding transplants).

Definitions of terms and key concepts described in this study protocol follow those proposed by the OMERACT initiative [9] and the Core Outcome Measures in Effectiveness Trials (COMET) Initiative [8]. They are presented in Table 2.

**Table 2:** Definitions of key concepts used in this study protocol (adapted from Boers et al. [9])

Key Concept	Definition
Health condition	A situation of impaired health
Health intervention	An activity performed by, for, with or on behalf of a client(s) whose purpose is to improve individual or population health, to alter or diagnose the course of a health condition or to improve functioning
Core area	An aspect of health or a health condition that needs to be measured to assess appropriately the effects of a health intervention (core areas are broad concepts consisting of a number of more specific concepts called domains)
Domain	Component of core area: a concept to be measured, a further specification of an aspect of health, categorised within a core area
Outcome	Any identified result in a domain arising from exposure to a casual factor or a health intervention
Measurement instrument	A tool to measure a quality or quantity of a variable; in this context, a domain or a contextual factor
Core domain set	In the study of health interventions, the minimum set of domains and subdomains necessary to cover adequately all core areas (fully measure all relevant concepts of a specific health condition within a specified scope); it describes what to measure
Core outcome measurement set	Definition introduced by the OMERACT Initiative
	The minimum set of outcome measurement instruments that must be administered in each intervention study of a certain health condition within a specified setting to cover adequately a corresponding core domain set; it describes how to measure
Core outcome set	Definition introduced by the COMET Initiative
	The agreed minimum set of outcomes that should be measured and reported in all clinical trials for a specific clinical area
Scope	The set of factors that describe the studies and circumstances to which the COS will apply; this is determined by the study questions and includes the health condition(s), target population, interventions and so forth
Contextual factor	A variable that is not an outcome of the study, but needs to be recognised (and measured) to understand the study results; this includes potential confounders and effect modifiers

### Aims and objectives

The aim of this study is to develop a COS relevant to all types of cardiac surgery effectiveness trials. The specific study objectives are to determine which outcome domains should be measured in all clinical trials (*what* to measure, the core domain set) and secondly, to define measurement instruments for the outcome domains (*how* to measure, the COS).

This does not imply that primary outcomes of cardiothoracic surgical trials should always and exclusively be those of the COS. However, to ensure the comparability of results across trials, the outcomes included in this COS should be considered for inclusion besides measuring trial-specific clinical endpoints.

## Methods/design

The conduct and reporting of this COS adheres, as much as is practicable, to the recommendations given by the COMET Initiative [8] on the development of a minimum COS in general, the methodological guidance provided by Sinha and colleagues [14] on the conduct and reporting of Delphi studies and by the OMERACT Initiative [9] on identifying outcome measurement instruments.

Ethical approval was obtained from the responsible ethics committee of the Faculty of Medicine, RWTH Aachen (EK 338/14).

### Scope of this COS

This COS is intended for clinical trials measuring the effectiveness of pre-, intra- or post-surgical interventions in non-minimally invasive off- or on-pump cardiothoracic surgery (elective and emergency procedures, excluding transplants, participants >18 years).

### Identification of existing knowledge

In preparation for this study, our group performed a systematic review of reviews to evaluate current clinical research on non-minimally invasive off- or on-pump cardiothoracic surgery (elective and emergency surgeries, excluding transplants and investigating pre-, intra- or post-surgical interventions) to determine the type and number of outcomes reported so far [4]. Pre-, intra- and post-surgical interventions were defined as any intervention related to the outcome of the procedure that occurred before, during or after cardiac surgery. Furthermore, we assessed to what extent outcomes in cardiothoracic surgical interventional trials were patient-centred and reflect patients’ perception, interpretation or evaluation of their condition and quality of care. Special focus was laid on endpoints that concentrated on salutogenesis, and 15 systematic reviews involving 371 randomised trials and 58,253 patients were included in our systematic review. We established unique lists of salutogenically and non-salutogenically focused outcomes, which collapsed into 38 outcome categories providing a list of potential core domains for the proposed minimum COS.

### Method to reach consensus on the core domain set (what to measure)

The Delphi method is iterative and uses a series of rounds of data collection and analysis to condense the opinions of individuals into a group consensus. Typically, it involves the use of sequential rounds of postal questionnaires that are designed to elicit participants’ opinions on a particular topic. Responses to each round are collated, analysed and redistributed to participants for further comment in successive rounds. Delphi surveys have been applied in other COS research groups [14]. Sinha and colleagues summarised key points, providing guidance on the Delphi technique to reach consensus on a minimum COS. Additionally, a recommended checklist of reporting items supports the reporting of Delphi studies, which we will adopt. Based on this, we decided to conduct an eDelphi survey online, to facilitate international participation without the time lag between successive rounds associated with traditional postal surveys, to enable a relatively low cost structure, to increase data collection efficiency and to provide the potential for a higher response rate through rapid communication with participants. The survey will be conducted using the online survey software QuestionPro (http://www.questionpro.com).

It is intended to reach consensus on core outcome domains (*what* to measure) in a three-round eDelphi exercise. The first round will contain all outcome domains identified by the aforesaid systematic review.

### Stakeholder involvement

Participation is sought from people within the following broad groups: adult patients in need of or after cardiothoracic surgery (any non-minimally invasive off- or on-pump operational procedure on the adult heart), cardiothoracic surgeons, cardiologists, anaesthesiologists, nursing staff involved with adult cardiothoracic patients and researchers with expertise in this particular field of medical research. There is currently no standard method for sample size calculations in Delphi processes, and thus a broad approach is taken to ensure there is sufficient and international participation in this Delphi study.

An email inviting participation will be sent to the following groups identified as relevant to the broad area of expertise regarding the subject under investigation: the German Heart Foundation, the British Cardiac Patients Association, the European Heart Network, the Support Network of the American Heart Association, the German Society for Thoracic and Cardiovascular Surgery (DGTHG), the European Association for Cardio-Thoracic Surgery, the American Association for Thoracic Surgery, the Society of Thoracic Surgeons, the Cardiothoracic Surgery Network (CTSNet), the German Society of Anaesthesiology and Intensive Care Medicine, the European Society of Anaesthesiology, the American Society of Anaesthesiologists and the Cochrane Heart Group. The aim is to approach researchers with knowledge of performing a meta-analysis and in a developing COS. Participants are then invited to use snowball sampling by forwarding the invitation to colleagues whom they regard as having the required expertise to contribute substantially to this eDelphi survey and the development of the COS. Those who participate will be asked to respond with their name, country of origin and email address. Prior to the eDelphi questions in round one, informed consent will be obtained and participants will be given information about the study and minimum COS. Here, plain language summaries provided by the COMET Initiative on COSs and the Delphi process in general will be utilised. These will provide patient representatives and professionals with the same information written in language suitable for all involved stakeholders. In addition, OMERACT Filter 2.0 (Table 2) will be presented to all participants highlighting especially the four core areas (death, life impact, resource use and pathophysiological manifestations), which should be addressed by the domains of every COS. However, our study group decided that we would not initially group the outcome domains identified via the systematic review according to the recommended core domains of OMERACT Filter 2.0. Considering the most commonly assessed domains in previous studies (measures of mortality, cerebrovascular complications and hospitalisation), we felt that such an approach would restrict the course of the eDelphi unjustifiably and that we would prejudge the selection of outcome domains for the core set.

Participants will also be asked to provide information on their educational and professional background, their experience with clinical research relevant for cardiac surgery and whether they were invited to participate as patients. Participants will be encouraged to complete the eDelphi questionnaire in each round. Each round will have a response closing date 28 days after the date of invitation. Email reminders will be send to anyone who did not respond by day 7 and after 2 weeks.

### eDelphi round 1

In the eDelphi, the order of potential core domains identified a priori via our systematic review of reviews will be randomised before being shown to participants. Based on the example given by Chiarotto et al. [15], participants will be asked to indicate if a domain is important enough to be included as a core domain. Response options will be: (a) “yes”, (b) “no” and (c) “unsure/I do not know”. Participants will be encouraged to suggest modifications of definitions and the wording of the domains. They will be asked to indicate if they consider that there is a large conceptual overlap between some domains and to judge whether some domains should be combined. Participants will also be asked to identify up to two new outcome domains, which they judge to be relevant or important.

The responses of round one will then be analysed using descriptive statistics. Frequencies on the importance of domains will be calculated for the whole panel. In addition, we will analyse responses from patient representatives separately to assess if they contrast from the general panel rating to ensure that their voice is heard. Our study team established a priori that domains for which at least 60 % of the participants choose the response option “no” and less than 20 % choose the response option “yes” will be dropped from the list of potential core domains. Additional outcome domains identified by participants in round one will be included if suggested by at least two participants.

### eDelphi round 2

A feedback report will be provided to the panel members before round 2 of the eDelphi. At this stage, the study team will propose deleting all domains from the list of potential core domains for which at least 60 % of the participants chose the response option “no” and less than 20 % chose the response option “yes”. Secondly, the study team will provide additional proposals based on participants’ comments from round one concerning, for example, the aggregation of other potential core domains. Participants will be asked to re-rate them at this point using their knowledge of their individual and the group’s previous ratings and to indicate whether they agree with the proposed decisions. The study team set consensus at 67 % agreement (two-thirds majority). Subsequently, participants will be asked to rate the newly identified outcome domains from round one using the same response options from round one: (a) “yes”, (b) “no” and (c) “unsure/I do not know”.

Upon completion, responses from round two will be analysed descriptively and summarised in a feedback report. Again, responses from patients’ representatives will be analysed separately to highlight deviations from the rest of the panel.

### eDelphi round three

In round three, participants who responded to round two will be presented with the feedback report from round two. Outcome domains retained after analysis of responses from round two will be presented and panel members will be asked if each outcome domain was indeed core. Response options will be the same as in the first two rounds and participants will be given the opportunity to provide arguments for their choices. As in round two, consensus will be set at 67 % for the panel to agree on the core domains to be included in the core domain set. If discrepancies arise or controversial arguments continue, the results will be discussed in depth within our study group, where the final decision will be made.

### Result of the eDelphi

The end result of the three-round eDelphi exercise will be the core domain set for all clinical trials investigating the effectiveness of pre-, intra- or post-surgical interventions in non-minimally invasive off- or on-pump cardiothoracic surgery (elective and emergency procedures, excluding transplants, participants >18 years). It is intended that the identified core domains should at best cover all four core areas (death, life impact, resource use and pathophysiological manifestations) of OMERACT Filter 2.0 (Table 1).

### Method to reach consensus on the COS (how to measure)

The final step in developing the anticipated COS will be to decide how to measure the identified core domains. For this step, we will follow the recommendations given by the OMERACT Initiative as described in the OMERACT Handbook [16]. The results of our systematic review of reviews will at best provide a list of potential measurement instruments; where no or no adequate list of possible instruments is available, a literature review will be performed to identify possible matching measurement instruments for each of the agreed core domains. Where only partially validated instruments are identified for the setting of the domains, or where no instruments are available in a domain, instruments will need to be further validated, or respectively developed, and their applicability documented.

Where no instruments are available, these need to be developed. For applicability, each instrument must prove to be valid, discriminative and feasible [9]. When all core domains can be measured by at least one applicable instrument, the end result is a draft that again is subjected to a consensus procedure with all stakeholders, resulting in the final COS.

### Implementation and updating of this COS

The uptake and implementation of a COS also needs to be carefully considered. An observational review [17] in a different clinical field (rheumatoid arthritis) was carried out to investigate whether there were trends in the proportion of trials reporting on the full set of core outcomes over time. The results suggest that a higher percentage of trialists conducting trials in rheumatoid arthritis are now measuring the rheumatoid arthritis COS. Therefore, COSs have the potential to improve the evidence base for a special clinical field, but consideration must be given to the methods for disseminating their availability amongst relevant stakeholders.

First and foremost, our COS is registered with the COMET database (http://www.comet-initiative.org/studies/details/630?result=true), which provides access to trialists and researchers involved in this clinical area.

To increase COS uptake, it is recommended [8] that developers consider engagement with the relevant Cochrane Review Groups, clinical guideline developers, research funders, journal editors, regulators such as research ethics committees, and trial registries. We intend to do so in close cooperation with the COMET Initiative. In addition, this COS and its development process will be introduced at a COMET meeting and at least at one national and international conference with a focus on cardiac surgery.

Reviewing a COS regularly is important as a form of validation, to ensure outcomes are still important to all stakeholders, to give the chance to add new outcomes, to evaluate how successful implementation has been and to engage further stakeholders if suitable [8]. The question of who should review a COS in what timeframes needs careful consideration. So far, no recommendations exist. Our study group will revisit this issue regularly and follow up on this topic within 2 years of the first published version of the proposed COS.

## Discussion

There is currently no COS relevant for clinical trials measuring the effectiveness of interventions in non-minimally invasive off- or on-pump cardiothoracic surgery. The proposed COS aims to improve cardiac interventional research by limiting reporting bias and heterogeneity across trials to ensure the comparability of effects and synthesis of research results in the future. We will involve multiple stakeholders and apply agreed qualitative standards to ensure the applicability and dissemination of the intended COS.

This research will benefit all stakeholders involved in cardiac surgical management in Germany, Europe and beyond. Researchers will be able to design clinical trials and synthesise research evidence that takes those outcomes under consideration that are important to all stakeholders. Thus, clinicians will be better equipped to facilitate informed decision-making by cardiac patients.

## Trial status

The eDelphi study started in March 2015. We intend to publish the results of the eDelphi, the core domain set, in late 2015.

## Abbreviations

COMET: Core Outcome Measures in Effectiveness Trials Initiative
COS: core outcome set
CTSNet: Cardiothoracic Surgery Network
CVD: cardiovascular disease
DGTHG: German Society for Thoracic and Cardiovascular Surgery
OMERACT: Outcome Measures in Rheumatology Initiative
